# A cross-sectional study on the correlation between exercise frequency and mental health among university students based on international physical activity questionnaire-short form classification: evidence from undergraduate students aged 18–22

**DOI:** 10.3389/fpsyg.2025.1616970

**Published:** 2025-07-15

**Authors:** Xingbin Du, Fugao Jiang, Shuping Yuan

**Affiliations:** ^1^College of General Education, Shandong Huayu University of Technology, Dezhou, China; ^2^School of Sports Science, Qufu Normal University, Qufu, China; ^3^Adult Education Office, Dongying Vocational and Technical College, Dongying, China

**Keywords:** university students, exercise frequency, mental health, depression, anxiety, international physical activity questionnaire-short form

## Abstract

**Background:**

Physical inactivity is tightly linked to mental health issues. In China, insufficient physical activity is common among university students, and their mental health concerns guarantee critical focus.

**Objective:**

Our study aims to conduct a cross-sectional investigation to explore the correlation between exercise frequency and mental health among university students, grouped according to the International Physical Activity Questionnaire (IPAQ). It further assesses differences in depressive and anxiety symptoms across groups with different levels of physical activity, to explain the protective influences of exercise on mental health.

**Methods:**

A cross-sectional design was adopted, including 430 undergraduate students aged 18–22 from Qufu Normal University and Shandong Huayu University of Technology. Participants were grouped into four groups according to the short form of the IPAQ (IPAQ-SF): frequent exercise group (FEG), occasional exercise group (OEG), infrequent exercise group (IEG), and no exercise group (NEG). Depressive and anxiety symptoms were evaluated utilizing the Patient Health Questionnaire-9 (PHQ-9) and the Generalized Anxiety Disorder-7 (GAD-7), in turn. Interfering factors including gender, grade level, body mass index (BMI), and sleep quality were managed for. One-way ANOVA and multiple logistic regression analyses were performed.

**Results:**

The NEG group displayed significantly higher scores for both depression (PHQ-9: 9.12 ± 4.05 vs. 4.62 ± 2.71) and anxiety (GAD-7: 8.54 ± 3.76 vs. 3.85 ± 2.29) compared with the FEG group (*p* < 0.001). Following adjustment, the risk of depression (OR = 7.85, 95% CI: 3.39–18.17) and anxiety (OR = 6.95, 95% CI: 2.83–17.05) sustained significantly promoted in the NEG group. Sleep quality also differed significantly in the subgroup of groups (*p* = 0.014), but sensitivity analyses revealed the robustness of the findings.

**Conclusion:**

There is a dose–response link between exercise frequency and mental health. Regular physical activity has a significant protective influence against depression and anxiety among university students. Universities are proposed to promote exercise-based intervention strageties to increase students’ mental well-being.

## 1 Introduction

Physical inactivity has become a significant public health issue globally, with a growing body of evidence indicating its detrimental effects on both physical and mental health (Al Nawaiseh et al., 2022). Based on the World Health Organization (WHO), by 2022, nearly one-third (31%) of the world’s adults did not meet the recommended levels of physical activity (Bai et al., 2024). This lack of physical activity not only enhances the risk of non-communicable diseases, such as cardiovascular and metabolic disorders, but it is also a well-documented modifiable risk factor for mental health problems, including depression and anxiety (Becker et al., 2017). These psychological outcomes have been consistently linked to physical inactivity across different population groups, with university students being a particularly vulnerable demographic (Deng et al., 2024).

The transition from adolescence to adulthood, which typically occurs during the university years, is a critical period for mental health (Field et al., 2005). During this phase, students face heightened stressors, such as academic pressure, social adjustments, and future uncertainties, which may predispose them to mental health disorders (Fluetsch et al., 2019). Depression and anxiety are among the most prevalent psychological issues within this group, with studies reporting that approximately 30–40% of university students globally suffer from some form of mental illness, commonly manifesting as symptoms of depression and anxiety (Deng et al., 2024). The high prevalence of mental health concerns in this population has profound implications for both their quality of life and academic performance, necessitating urgent attention to the factors influencing these conditions (Ghrouz et al., 2019).

The relationship between physical inactivity and mental health problems, particularly depression and anxiety, is well-established in recent literature (Goodwin, 2003; Grasdalsmoen et al., 2020). Theories of stress, such as the Transactional Model of Stress and Coping, suggest that physical activity may mitigate stress by enhancing psychological resilience and reducing the physiological markers of stress, such as cortisol levels, which are commonly elevated in individuals with depression and anxiety (Harber and Sutton, 1984). Furthermore, Biopsychosocial Models of health propose that regular physical activity triggers physiological changes, including the release of neurotransmitters like serotonin and endorphins, which are known to promote mood and reduce anxiety symptoms (Ibrahim et al., 2013). These theories suggest that physical activity could act as an effective buffer against the adverse psychological effects associated with academic stress and other challenges faced by university students.

Despite the well-documented link between physical activity and mental health, particularly in relation to depression and anxiety, much of the recent research has been confined to specific contexts, such as during the COVID-19 pandemic or within limited demographic groups (Jayakody et al., 2014; Kljajević et al., 2021). Few studies have explored the relationship between different frequencies of physical activity and mental health in the general university population under normal circumstances (Koenig et al., 2025; Lee et al., 2012). Additionally, recent evidence commonly fail to account for key confounding factors, such as sleep quality, body mass index (BMI), and academic stress, which may also influence mental health outcomes (Li et al., 2024; Mammen and Faulkner, 2013).

The aim of this study is to fill this gap by exploring the relationship between exercise frequency and mental health outcomes, specifically depression and anxiety, among a cohort of university students aged 18–22 years. We hypothesize that higher levels of physical activity, as measured by frequency, will be associated with lower levels of depression and anxiety. This cross-sectional study includes students from Qufu Normal University and Shandong Huayu University of Technology in China and uses a validated set of mental health assessment tools, including the Patient Health Questionnaire-9 (PHQ-9) and the Generalized Anxiety Disorder-7 (GAD-7). Besides, we will adjust for potential confounding factors, such as gender, BMI, academic year, and sleep quality, to provide a more robust and comprehensive understanding of the relationship between physical activity and mental health in this population.

## 2 Materials and methods

### 2.1 Research design and subjects

Our study is a cross-sectional study. The research subjects are undergraduate students aged 18–22 years old at Qufu Normal University and Shandong Huayu University of Technology (set in Qufu City, Shandong Province, China). Our study was conducted at this university from March to May 2024. The approach of graded cluster sampling was used to randomly select classes from the first to the fourth grades and invite all students in these classes to take part in the questionnaire survey. The inclusion criteria are: aged 18–22 years old, recently signed up for this university, and keen on take part in the study. The exclusion criteria include: those who refused to offer well-informed consent; those with serious physical diseases that prevent them from taking part in daily exercise generally; those who have been previously diagnosed with severe mental disorders (such as schizophrenia, severe depression, etc.) that influence their self-assessment of mental health; and those with incomplete questionnaire information. Based on the sample size plan devised in advance by the research team, totally 500 questionnaires were issued in our study, and 452 valid questionnaires were truly collected (the positive response rate was 90.4%). Following excluding those who failed to meet the inclusion and exclusion criteria, totally N = 430 subjects were lastly included in the assessment. This sample size meets the minimum sample number essential for the previous sample size estimation and has sufficient statistical power (see the sample size estimation below).

### 2.2 Grouping and evaluation of exercise frequency

The physical activity status of the subjects was gained through a standardized questionnaire survey. We used the Chinese version of the International Physical Activity Questionnaire-Short Form (IPAQ-SF) to assess exercise frequency and exercise volume (Fluetsch et al., 2019). The IPAQ-SF was advanced under the leadership of the WHO and is used to collect information on physical activities in the past 7 days, including the number of days and time of moderate-and high-intensity exercises, along with the amount of walking activities. The Chinese version of this questionnaire has been verified to have good reliability and validity in the Chinese population (Fluetsch et al., 2019). Based on the data gained from the IPAQ-SF, we divided the subjects into four groups based on their weekly exercise frequency: (1) Frequent exercise group (FEG): those who took part in moderate or above-intensity exercise ≥ 3 days per week; (2) Occasional exercise group (OEG): those who exercised 1–2 days per week; (3) Infrequent exercise group (IEG): those who exercised less than 1 day per week on average (but not fully without exercise, that is, there were signs of occasional exercise within the recent week); (4) No exercise group (NEG): those who failed to take part in any moderate-or high-intensity exercise within the recent week. The above grouping is according to a comprehensive reference to the proposed amount of physical activity for adults by the WHO and the classification stragetes in current literatures. For each subject, we also calculated the metabolic equivalent (MET) minutes of their total weekly physical activity according to the IPAQ-SF, but the above exercise frequency grouping was primarily used for the independent variable analysis. Throughout the questionnaire survey, we asked the subjects to honestly report their exercise status and offered unified instructions and examples to confirm consistent understanding.

### 2.3 Mental health assessment

In our study, only standardized psychological assessment scales were used to evaluate the mental health status of university students. All scales used current Chinese translations and have been indicated to have good reliability and validity in the Chinese youth population. The assessment of depressive symptoms used the Chinese version of the Patient Health Questionnaire-9 (PHQ-9; Harber and Sutton, 1984). The PHQ-9 is a extensively used self-report scale for screening and evaluating the severity of depressive symptoms in the past 2 weeks, and it has been indicated to have good reliability and validity in the subgroup of Chinese university students (Harber and Sutton, 1984).

The assessment of anxiety symptoms used the Chinese version of the Generalized Anxiety Disorder 7-item scale (GAD-7; Ibrahim et al., 2013). The GAD-7 is used to assess the frequency of the core symptoms of generalized anxiety disorder, and the Chinese version of this scale has also been indicated to have good psychometric properties in the Chinese youth population (Ibrahim et al., 2013). Both of the above scales were self-completed by the subjects, and the total scores were calculated in turn (the total score range of PHQ-9 is 0–27 points, and the total score of GAD-7 is 0–21 points). The higher the score, the more severe the depressive or anxiety symptoms. We used the identified cut-off significances to assist in recognizing the clinically significant psychological symptoms: a PHQ-9 score ≥ 10 points was used as the cut-off significance for moderate and above depressive symptoms (Meeusen and De Meirleir, 1995), and a GAD-7 score ≥ 10 points was used as the cut-off significance for moderate and above anxiety symptoms. In addition to depression and anxiety, the research questionnaire also included self-assessment content of general mental health status, such as a single-item assessment of one’s own psychological stress and subjective well-being (utilizing a 0–10 rating scale) to offer a supplementary description of the overall mental health of the subjects. The selection and utilize of these assessment tools seek to comprehensively and objectively reflect the mental health level of university students.

### 2.4 Collection of interfering variables and other information

To control the influence of potential interfering factors, Our study also obtained information on the demographic characteristics and health-related behaviors of the subjects. Basic demographic data, such as gender (male/female), actual age, and grade (from the first to the fourth year of university) were gained through self-completion of the questionnaire. The subjects displayed their height (cm) and weight (kg), and the researchers calculated the body mass index (BMI, kg/m^2^) accordingly. The sleep quality was evaluated utilizing the Chinese version of the Pittsburgh Sleep Quality Index (PSQI) scale. The PSQI includes 7 components (subjective sleep quality, sleep latency, sleep duration, sleep efficiency, sleep disturbances, utilize of sleeping medications, and daytime dysfunction), with a total score of 0–21 points. The higher the score, the worse the sleep quality. This scale has been extensively used in China and has been indicated to have good reliability and validity for the assessment of sleep in the youth population. We recorded the total PSQI score of each subject and used it as a continuous covariate to reflect the sleep status in the analysis. Besides, the questionnaire also asked about other potentially related personal information, such as whether they smoke or drink, whether they have chronic diseases, and the family economic situation, etc., to describe the sample characteristics and consider the influence on the research results. In the data analysis, we primarily managed for the interfering factors pre-specified in the research design (such as gender, grade, BMI, sleep quality, etc.), and the remaining obtained information was used for descriptive statistics or sensitivity analysis as needed.

### 2.5 Sample size estimation

The sample size for this study was calculated based on a literature review and estimated effect size. We assumed a medium effect size (*f* = 0.25) for differences in mental health scores between exercise frequency groups, based on previous studies exploring similar relationships between physical activity and mental health in university student populations. To ensure robust statistical power, the calculation used G*Power 3.1 software with the following parameters: a significance level of *α* = 0.05, power of 0.80 (80% statistical power), and a medium effect size (f = 0.25). This resulted in a minimum sample size estimate of N = 280 for detecting significant differences with a one-way ANOVA. Considering a 20% response rate for non-responses or incomplete data, the target recruitment was increased to approximately N = 350. During data collection, we distributed 500 questionnaires, receiving 452 valid responses. After excluding 22 responses that either failed to meet eligibility criteria or lacked key data, the final analytic sample consisted of 430 participants. This sample size exceeded the minimum required (N = 280), ensuring over 80% statistical power at the 0.05 significance level, and provides sufficient power to detect significant differences across the exercise frequency groups.

### 2.6 Statistical analysis

All data were double-entered and analyzed using SPSS version 26.0. Descriptive statistics were used to summarize baseline characteristics and key variables, with continuous variables presented as mean ± standard deviation (SD) or median with interquartile range (IQR), depending on the normality of distribution. Categorical variables were presented as frequencies and percentages. For comparing demographic and mental health variables across exercise frequency groups, one-way ANOVA was used for normally distributed continuous variables, the Kruskal-Wallis test for non-normally distributed variables, and the chi-square (χ^2^) test for categorical variables. To evaluate the independent association between exercise frequency and mental health outcomes, multiple logistic regression analyses were performed. For continuous mental health scores (e.g., PHQ-9, GAD-7), multivariate linear regression models were used with FEG as the reference group, while for binary outcomes (e.g., presence of moderate-to-severe depressive or anxiety symptoms), multivariate logistic regression models were employed to calculate odds ratios (ORs). All regression models adjusted for pre-specified covariates including gender, academic year, BMI, and sleep quality. The results of the linear regression are presented as regression coefficients with 95% confidence intervals (CIs), while the results of logistic regression are presented as ORs with 95% CIs. A significance level of *p* < 0.05 was considered statistically significant. To account for multiple comparisons across the four exercise frequency groups, Bonferroni correction was applied by dividing the original significance level (α = 0.05) by the number of comparisons (3), resulting in a corrected significance level of 0.0167 for each pairwise comparison, thus controlling the Type I error rate and ensuring the robustness of the findings. Besides, a sensitivity analysis was conducted by excluding participants with poor sleep quality (PSQI > 10), with the aim of evaluating the influence of sleep quality on the uncovered relationships between exercise frequency and mental health outcomes. This analysis confirmed that the main findings remained consistent, further supporting the validity of the results.

### 2.7 Ethical considerations

Our study was approved by the Medical Ethics Committee of Qufu Normal University (Approval No. 2024175) and Shandong Huayu University of Technology (Approval No. 2024096) and was performed in line with the principles of the Declaration of Helsinki. Before participation, all subjects were well-informed of the study’s purpose, and procedures, and offered written well-informed consent. Participation was fully voluntary, and respondents were authorized to withdraw at any time without consequence. All data were obtained anonymously utilizing coded questionnaires, and only the research team had access to raw data. Results were used only for academic research purposes. A pre-established referral protocol was in place for participants exhibiting signs of psychological distress or health risks, determining they attracted appropriate counseling or medical confirm. Our study adhered harshly to ethical standards and regulations, determining maximal protection of participants’ rights and privacy.

## 3 Results

### 3.1 Baseline characteristics of participants

Totally 430 valid responses were included in the final analysis. The mean age of participants was 20.13 ± 1.16 years, with 182 males (42.3%) and 248 females (57.7%). The distribution across academic years was relatively balanced: 109 (25.3%) first-year, 112 (26.0%) second-year, 105 (24.4%) third-year, and 104 (24.2%) fourth-year students. The average BMI was 21.36 ± 2.79 kg/m^2^, and 78.6% of the participants had BMI significances within the reference range (18.5–23.9 kg/m^2^). The mean PSQI score was 6.21 ± 2.58, determining that most participants had fair sleep quality. Based on IPAQ-SF classification, participants were grouped as follows: 112 (26.0%) in the FEG, 126 (29.3%) in OEG, 108 (25.1%) in the IEG, and 84 (19.5%) in the NEG. There were no significant differences in the subgroup of the four groups in regard to gender, academic year, or BMI (*p* > 0.05). However, sleep quality differed significantly across groups (*p* = 0.014), with the NEG reporting the poorest sleep. Table 1 shows the baseline characteristics.

**Table 1:** Baseline characteristics of participants.

Variable	FEG	OEG	IEG	NEG	*p*-value
Sample Size (*n*)	112 (26.0%)	126 (29.3%)	108 (25.1%)	84 (19.5%)	–
Age (years)	20.11 ± 1.13	20.15 ± 1.18	20.12 ± 1.15	20.16 ± 1.19	0.982
Gender (M/F)	48/64	54/72	42/66	38/46	0.895
Academic Year (1/2/3/4)	28/29/27/28	30/33/30/33	27/28/27/26	24/22/21/17	0.987
BMI (kg/m^2^)	21.25 ± 2.68	21.43 ± 2.81	21.38 ± 2.75	21.49 ± 2.89	0.965
Sleep Quality (PSQI)	5.89 ± 2.43	6.12 ± 2.55	6.35 ± 2.61	6.78 ± 2.72	0.014*

### 3.2 Mental health status across exercise frequency groups

Significant differences were uncovered in both PHQ-9 and GAD-7 scores across the exercise frequency groups. The mean PHQ-9 score in the Frequent Exercise Group (FEG) was 4.62 ± 2.71, significantly lower than that of the Occasional Exercise Group (OEG; 6.35 ± 3.10), Infrequent Exercise Group (IEG; 7.68 ± 3.42), and No Exercise Group (NEG; 9.12 ± 4.05), with a statistically significant overall difference (*F* = 28.62, *p* < 0.001). After applying the Bonferroni correction, the adjusted significance level for pairwise comparisons was set at 0.0167. Bonferroni-adjusted pairwise comparisons revealed the following: FEG differed significantly from both IEG and NEG (*p* < 0.001 for both comparisons), OEG also differed significantly from NEG (*p* = 0.006). Similarly, for GAD-7 scores, the FEG had the lowest mean score of 3.85 ± 2.29, which was significantly lower than those of the OEG (5.21 ± 2.97), IEG (6.93 ± 3.34), and NEG (8.54 ± 3.76), with a significant overall group difference (*F* = 34.19, *p* < 0.001). After applying the Bonferroni correction, pairwise comparisons showed: FEG differed significantly from both IEG and NEG (*p* < 0.001 for both comparisons), OEG also differed significantly from NEG (*p* = 0.008). In terms of clinical symptom prevalence, using a PHQ-9 score ≥ 10 as the threshold for moderate-to-severe depressive symptoms, the FEG group had a prevalence of 6.3%, significantly lower than the OEG group (14.3%), IEG group (22.2%), and NEG group (33.3%; χ^2^ = 26.51, *p* < 0.001). After Bonferroni correction, the significance remained for comparisons between FEG vs. IEG (*p* < 0.001) and FEG vs. NEG (*p* < 0.001). For anxiety, using a GAD-7 score ≥ 10 to define moderate-to-severe anxiety, the FEG group had a prevalence of 4.5%, compared to 11.1% in the OEG, 18.5% in the IEG, and 28.6% in the NEG (χ^2^ = 24.73, *p* < 0.001). After Bonferroni correction, significant differences remained between FEG and NEG (*p* < 0.001) and OEG and NEG (p = 0.008; Table 2).

**Table 2:** Differences in depression and anxiety across exercise frequency groups.

Variable	FEG (*n* = 112)	OEG (*n* = 126)	IEG (*n* = 108)	NEG (*n* = 84)	Test statistic	*p*-value	Bonferroni adjusted *p*-value
PHQ-9 Score	4.62 ± 2.71	6.35 ± 3.10	7.68 ± 3.42	9.12 ± 4.05	F = 28.62	<0.001	<0.001
GAD-7 Score	3.85 ± 2.29	5.21 ± 2.97	6.93 ± 3.34	8.54 ± 3.76	F = 34.19	<0.001	<0.001
PHQ-9 ≥ 10 (*n*, %)	7 (6.3%)	18 (14.3%)	24 (22.2%)	28 (33.3%)	χ^2^ = 26.51	<0.001	<0.001
GAD-7 ≥ 10 (*n*, %)	5 (4.5%)	14 (11.1%)	20 (18.5%)	24 (28.6%)	χ^2^ = 24.73	<0.001	<0.001

### 3.3 Multiple logistic regression analyses adjusted for confounders

After controlling for gender, academic year, BMI, and sleep quality, multivariate linear regression displayed significantly higher PHQ-9 scores in OEG (*β* = 1.52, 95% CI: 0.72–2.32, *p* < 0.001), IEG (β = 3.09, 95% CI: 2.25–3.93, *p* < 0.001), and NEG (β = 4.47, 95% CI: 3.54–5.40, *p* < 0.001) compared with FEG. Equally, GAD-7 scores were significantly promoted in OEG (β = 1.22, 95% CI: 0.54–1.90, *p* = 0.001), IEG (β = 2.83, 95% CI: 2.10–3.56, *p* < 0.001), and NEG (β = 4.13, 95% CI: 3.31–4.95, *p* < 0.001) compared with FEG. Multivariate logistic regression displayed the highest risk of moderate-to-severe depressive symptoms in the NEG (adjusted OR = 7.85, 95% CI: 3.39–18.17, *p* < 0.001), followed by the IEG (adjusted OR = 4.27, 95% CI: 1.85–9.84, *p* = 0.001). Equally, for anxiety, NEG displayed significantly increased risk (adjusted OR = 6.95, 95% CI: 2.83–17.05, *p* < 0.001), and IEG also displayed promoted risk (adjusted OR = 3.99, 95% CI: 1.61–9.90, *p* = 0.003). Table 3 shows the regression results.

**Table 3:** Multiple logistic regression analysis of the influence of exercise frequency on mental health.

Model Type	FEG (Ref.)	OEG	IEG	NEG
Linear Regression (PHQ-9)	–	β = 1.52 (0.72–2.32); *p < 0.001*	β = 3.09 (2.25–3.93); *p < 0.001*	β = 4.47 (3.54–5.40); *p < 0.001*
Linear Regression (GAD-7)	–	β = 1.22 (0.54–1.90); *P = 0.001*	β = 2.83 (2.10–3.56); *p < 0.001*	β = 4.13 (3.31–4.95); *p < 0.001*
Logistic Regression (Depression)	–	OR = 2.43 (1.02–5.80); *p = 0.045*	OR = 4.27 (1.85–9.84); *p = 0.001*	OR = 7.85 (3.39–18.17); *p < 0.001*
Logistic Regression (Anxiety)	–	OR = 2.25 (0.93–5.44); *p* = 0.071	OR = 3.99 (1.61–9.90); *p = 0.003*	OR = 6.95 (2.83–17.05); *p < 0.001*

### 3.4 Sensitivity analysis

To evaluate the robustness of the results, a sensitivity analysis was carried out by excluding participants with poor sleep quality (PSQI >10). The findings sustained consistent, with significant differences in mental health scores in the subgroup of exercise frequency groups (PHQ-9: *F* = 24.91, *p* < 0.001; GAD-7: *F* = 29.56, *p* < 0.001). Table 4 shows the sensitivity analysis results.

**Table 4:** Sensitivity analysis after excluding participants with poor sleep quality (PSQI > 10).

Variable	FEG (*n* = 105)	OEG (*n* = 119)	IEG (*n* = 101)	NEG (*n* = 78)	Test statistic	*p*-value
PHQ-9 Score	4.58 ± 2.69	6.28 ± 3.05	7.59 ± 3.37	9.01 ± 3.98	F = 24.91	<0.001*
GAD-7 Score	3.79 ± 2.25	5.15 ± 2.91	6.85 ± 3.28	8.42 ± 3.69	*F* = 29.56	<0.001*
PHQ-9 ≥ 10 (*n*, %)	6 (5.7%)	16 (13.4%)	22 (21.8%)	25 (32.1%)	χ^2^ = 24.12	<0.001*
GAD-7 ≥ 10 (*n*, %)	5 (4.8%)	13 (10.9%)	18 (17.8%)	21 (26.9%)	χ^2^ = 22.35	<0.001*

## 4 Discussion

### 4.1 Main findings of the study

This study establishes a significant and robust link between exercise frequency and mental health outcomes among university students. The data reveal a clear and progressive relationship: as exercise frequency decreases, the severity of depression and anxiety symptoms enhances significantly. In particular, students who engage in moderate or high-intensity exercise at least three times a week exhibit markedly lower mental health risks, as measured by both depression (PHQ-9) and anxiety (GAD-7) scores, when compared to those with minimal or no exercise. NEG, for example, displayed depression and anxiety scores almost twice as high as those of the FEG. These findings strongly suggest that regular physical activity serves as a protective factor for mental health, with those in the NEG reporting clinical symptoms of depression (33.3%) and anxiety (28.6%) at much higher rates than those in the FEG (6.3 and 4.5%, respectively).

Furthermore, after controlling for critical confounding factors such as sleep quality, gender, and BMI, the relationship between exercise frequency and mental health outcomes remained statistically significant and robust. The adjusted ORs for moderate-to-severe depressive and anxiety symptoms were found to be 7.85 and 6.95, respectively, for the NEG group compared to the FEG. These results underscore the protective role of exercise in the mental well-being of university students, particularly in mitigating the risks of depression and anxiety associated with physical inactivity.

This study’s findings are particularly important given the growing prevalence of mental health issues among university students, a demographic that faces unique challenges during this pivotal life stage. Our results suggest that interventions aimed at increasing physical activity could be an effective strategy to promote mental health and reduce the burden of mental disorders in this population.

### 4.2 Comparison with previous studies

While a body of literature has consistently displayed the protective effects of physical activity on mental health, this study offers several novel contributions that extend the recent knowledge base (Papalia et al., 2018). Previous research has commonly focused on specific populations or context-bound situations, such as the COVID-19 pandemic, which may limit the generalizability of findings (Pearce et al., 2022; Perillat and Baigrie, 2021). Conversely, our study, conducted in a typical campus environment, includes a diverse sample of Chinese university students and offers broader insights into the general effects of exercise on mental health. By utilizing the IPAQ-SF for precise classification of exercise frequency, this study provides a more accurate assessment of physical activity patterns compared to previous studies that may have counted on self-displayed or less standardized measures.

Moreover, while other studies have noted the association between lower levels of physical activity and poorer mental health (Perillat and Baigrie, 2021; Rebar et al., 2015), the large sample size and rigorous control for confounding variables in our study lend substantial strength to our findings. The application of multivariate regression analysis further promotes the validity of our results, allowing us to control for factors such as sleep quality, BMI, and academic year, which have been displayed to influence mental health outcomes. These features of our study make it more representative and generalizable, contributing to the body of evidence supporting the role of exercise in promoting mental health.

### 4.3 Potential physiological and psychological mechanisms

Our findings are supported by recent theoretical frameworks that suggest physical activity influences mental health through both physiological and psychological mechanisms. From a physiological standpoint, regular exercise is known to regulate neurotransmitter systems in the brain, including enhancing the release of serotonin, dopamine, and endorphins (Santos et al., 2023; Singh et al., 2023). These neurochemicals are crucial in regulating mood, alleviating anxiety, and reducing symptoms of depression (Santos et al., 2023; Singh et al., 2023). Specifically, the elevation of *β*-endorphins, commonly associated with the “runner’s high,” is strongly linked to mood promotement and psychological well-being (Spitzer et al., 2006). Additionally, physical activity has been displayed to lower cortisol levels and reduce the overactivation of the hypothalamic–pituitary–adrenal (HPA) axis, a response that is commonly seen in individuals experiencing chronic stress, depression, and anxiety (Sun et al., 2020). Regular exercise can thus help manage the physiological markers of stress, contributing to long-term mental health benefits (Ünal and Özenoğlu, 2025).

Psychologically, exercise has been displayed to promote self-esteem and self-efficacy, enhancing individuals’ perceptions of their capabilities and reducing feelings of helplessness or worthlessness commonly associated with depression (Xiang et al., 2020; Xue et al., 2025). Furthermore, exercise commonly promotes social interactions, which can increase social support and foster a sense of belonging—both of which have been linked to reduced levels of perceived stress and mental health disorders (Yim, 2016). The positive social and psychological benefits derived from physical activity further highlight its multifaceted role in improving mental well-being.

Additionally, our study suggests that sleep quality may act as an important mediating factor in the relationship between physical activity and mental health. We uncovered that participants who engaged in regular exercise displayed significantly better sleep quality, which in turn is associated with reduced symptoms of depression and anxiety. Exercise may promote sleep by shortening sleep latency, extending deep sleep phases, and reducing nocturnal awakenings (Zhang et al., 2021). These sleep promotements likely contribute to the promoted emotional stability uncovered in more physically active individuals, emphasizing the need for holistic mental health interventions that incorporate both physical activity and sleep hygiene.

### 4.4 Practical significance and intervention suggestions

The results of our study emphasize the significant role of physical activity in enhancing university students’ mental health. Based on our findings, we suggest that universities implement specific and measurable strategies to increase students’ physical activity levels and promote their mental well-being.

To ensure regular physical activity, we recommend that universities offer at least three moderate intensity exercise classes per week. This will help students engage in sustained and effective physical activities, resulting in better mental health outcomes. Universities can integrate technology into their health promotion plans by developing mobile applications or using recent applications to monitor students’ exercise frequency. Research has displayed that interventions for college students using smartphone applications to track their exercise levels have been proven to help promote their physical activity levels. For example, a randomized controlled trial showed that the intervention group successfully promoted students’ physical activity by setting daily step goals through the use of a mobile health application (Zhang et al., 2025). Besides, another study found that the most commonly used exercise monitoring tool among college students is a mobile based application (Zhang et al., 2013). These research results indicate that mobile technology can be effectively integrated into health promotion programs, helping students increase their exercise frequency and promote their health awareness. Based on a systematic review study, the promotement of physical activity and fitness among college students can be achieved through activity registration through an application to ensure that students reach the recommended level of activity (Wei et al., 2024).

In addition, the creation of campus sports culture, such as promoting students’ social, academic, physical, and psychological development through regular extracurricular sports activities (OEP), also helps to increase students’ enthusiasm for participating in sports activities (Hossain et al., 2024). These studies indicate that utilizing technological means for activity tracking and encouraging sports participation through campus culture can effectively promote students’ physical health and engagement. Universities can provide personalized exercise plans for students based on their individual health levels. This tailored approach will meet students’ unique physical abilities and mental health needs, thereby encouraging more people to participate in sports activities. To further promote the benefits of exercise for mental health, universities can combine mental health support programs with physical activities. For example, creating stress relieving exercise classes, such as yoga or mindfulness based exercise classes, can help students cope with academic stress, reduce anxiety and depression.

By implementing these specific strategies, universities can foster an environment where students are more likely to engage in regular physical activity, ultimately improving their mental health and well-being. These practical measures provide universities with clear, actionable steps to promote students’ overall health.

### 4.5 Advantages and innovations of the study

This study has several key advantages that promote its academic value. Interestingly, the use of the IPAQ-SF provides an accurate and reliable measure of physical activity levels, allowing for precise classification of participants into exercise frequency groups. It is worth noting that our study is based on a large, diverse sample of university students, which enhances its generalizability. Furthermore, we employed rigorous statistical methods, including multivariate regression analysis, to control for confounding variables, ensuring the robustness of our findings. These advantages make this study one of the most comprehensive investigations into the correlation between exercise frequency and mental health among university students, especially within the Chinese context. The findings provide a strong scientific basis for developing targeted mental health policies and interventions aimed at improving student well-being.

### 4.6 Limitations of the study

Despite its advantages, this study has certain limitations. The cross-sectional design precludes any conclusions about causality. Future research should adopt longitudinal or experimental designs to further elucidate the causal relationship between exercise and mental health. Additionally, our reliance on self-displayed data regarding physical activity may introduce recall bias or social desirability bias. Future studies should consider using more objective measures of physical activity, such as accelerometers or heart rate monitoring, to mitigate these biases. Furthermore, while we controlled for key confounding factors such as gender, academic year, BMI, and sleep quality, there may be other unmeasured variables, such as dietary habits and social support, that could influence the uncovered relationships. Future studies should aim to include these additional factors to provide a more comprehensive understanding of the relationship between physical activity and mental health. Another limitation of this study is the sample source, which was limited to students from two universities in Shandong Province. As such, the findings may not fully represent the national university student population. The sample’s regional focus limits the generalizability of the results to students from other regions or countries. Future research should aim to expand the sample coverage, incorporating participants from a broader range of universities across China and internationally to promote the external validity and generalizability of the findings.

### 4.7 Future research directions

This study is based on a cross-sectional design, which precludes the ability to establish causal relationships between physical activity and mental health outcomes. Therefore, longitudinal research is needed to verify the causal relationship between exercise and mental health in university students. Future studies should explore the long-term effects of physical activity on mental health by following participants over extended periods to uncover the changes and influences over time. Additionally, research should investigate the effects of different types (e.g., aerobic, resistance training) and intensities (e.g., moderate vs. vigorous) of physical activity on mental health outcomes. This would provide a clearer understanding of how specific exercise interventions might benefit mental health in the university student population.

Furthermore, incorporating neuroscientific and psychological approaches could help to uncover the mechanisms by which physical activity influences brain function, cognitive performance, and stress responses. By integrating these perspectives, future research can provide deeper insights into the complex relationship between physical activity and mental health, enabling more tailored interventions that could effectively promote the well-being of students.

## 5 Conclusion

We assess the correlation between different exercise frequencies and mental health in the context of a sample of Chinese university students. The findings displayed that the lower the frequency of physical activity, the higher the risks of depression and anxiety among university students, and this link remains significant after managing key variables, such as sleep quality, gender, and BMI. Our study stresses that regular moderate or above-intensity exercise is not only an positive path to strengthening the physical and mental health of university students, but also a significant public health strategy for avoiding mental concerns. Future research should be further deepened from the aspects of longitudinal tracking, mechanism analysis, and intervention promotement, and the integration of exercise interventions into the mental health service system of universities should be promoted to address the increasingly prominent mental health problems among university students.

## Data Availability

The raw data supporting the conclusions of this article will be made available by the authors, without undue reservation.
